# Impact of herpes zoster and postherpetic neuralgia on the quality of life of Germans aged 50 or above

**DOI:** 10.1186/s12879-018-3395-z

**Published:** 2018-10-03

**Authors:** Desmond Curran, Ruprecht Schmidt-Ott, Ulf Schutter, Jörg Simon, Anastassia Anastassopoulou, Sean Matthews

**Affiliations:** 1 Health Economics Department, GSK, Avenue Fleming 20, 1300 Wavre, Belgium; 2 Current address: Bavarian Nordic, Martinsried, Germany; 3 Facharztzentrum am Marienhospital und Marler Arztnetz, Marl, Germany; 4 Gesundheitsnetz Osthessen, Fulda, Germany; 5 GSK, Munich, Germany

**Keywords:** Herpes zoster, Postherpetic neuralgia, Zoster incidence, Pain, Activities of daily living, Quality of life

## Abstract

**Background:**

Herpes zoster (HZ) is a painful dermatomal rash caused by reactivation of latent varicella zoster virus surviving in the patient’s sensory ganglia after a previous episode of varicella. The incidence of HZ increases markedly with age as does the proportion of HZ patients who develop postherpetic neuralgia (PHN) with often severe and debilitating pain persisting for months and even years.

This prospective study aimed to assess the impact of HZ and PHN on the quality of life (QoL) of individuals aged ≥ 50 years in Germany.

**Methods:**

Patients were recruited when consulting primary care physicians for a first HZ episode. PHN was defined as a ‘worst’ pain score ≥ 3 on the Zoster Brief Pain Inventory (ZBPI) scale persisting or appearing 90 days or more after rash onset. PHN-cases were followed for up to nine months after rash onset. The interference of pain with patients’ ability to carry out normal activities was assessed by the ZBPI activities of daily living (ADL) scale and QoL by the EuroQoL five-dimension scale (EQ-5D) utility score.

**Results:**

Of 513 patients enrolled, 61 (11.9%) developed PHN. At HZ onset, the mean ZBPI worst pain score of all patients was 5.1, the least square (LS)means estimates of the ZBPI ADL and EQ-5D utility scores were 2.970 and 0.740, respectively. Over three months follow-up, the pain scores decreased and the QoL increased monotonically across all age groups. At Day 90, the mean ZBPI worst pain score of the PHN patients was 4.4, while the LSmeans estimates of the ZBPI ADL and EQ-5D utility scores were 2.899 and 0.826, respectively. For patients with PHN persisting at nine months, the pain scores and QoL remained unchanged over the six months following the development of PHN.

**Conclusion:**

HZ and PHN had a substantial impact on the patients’ QoL and ability to function in their normal activities. There was a clear association in time between the evolution of pain and estimated QoL. The impact on ADL and QoL did not vary with age.

**Electronic supplementary material:**

The online version of this article (10.1186/s12879-018-3395-z) contains supplementary material, which is available to authorized users.

## Background

Herpes zoster (HZ) is a painful and debilitating condition caused by a reactivation of varicella-zoster virus (VZV), which as a primary infection causes chickenpox [1, 2]. In the primary infection, the virus establishes life-long latency in multiple sensory ganglia and HZ is a secondary infection with spread of the virus, usually to a single dermatome [1, 2].

Acute HZ is usually characterized by a vesicular rash of the skin of the affected dermatome generally accompanied by acute pain [1–3]. Acute HZ is normally defined as lasting for up to 30 days after rash onset, which for patients developing long-term disease is followed by a subacute phase lasting 30–90 days from rash onset. Most patients experience non-specific symptoms like itching, burning sensation or general constitutional symptoms (fever, malaise for instance) in the days before rash onset, a phase called the prodrome [3].

The most common sequelae of HZ is postherpetic neuralgia (PHN) involving chronic pain that occurs or persists after the rash has resolved. The cause of PHN is not known [2] and various definitions of the syndrome are used as there is no universal agreement regarding pain intensity, type of pain and duration of pain required. Consequently, study reports of the proportion of HZ patients with PHN vary greatly [4]. The most commonly used definition of PHN is the presence of moderate or severe ‘worst’ pain (rated ≥ 3 on the zoster brief pain inventory (ZBPI) ‘worst pain’ scale) persisting or appearing 90 days or more after rash onset.

Without varicella vaccination programs virtually all inhabitants of temperate regions of the world will have had wild type VZV infection before the age of 30 years and are consequently at risk of developing HZ possibly followed by PHN [4]. Development of HZ is associated with decreasing VZV-specific cellular immunity, either due to natural immunosenescence related to aging or due to immunocompromise caused by disease or by immunosuppressant therapy [2, 4]. The incidence and severity of HZ thus increases markedly after the age of 50 years just as the risk of developing PHN [4].

It is increasingly recognized that HZ and PHN can lead to considerable impairments of the patients’ health related quality of life (QoL) [2, 5]. In particular, the acute and chronic pain may affect the patients’ activities of daily living (ADL), social interactions and psychological well-being [3] and may even reduce the patients’ ability to maintain an independent way of living [6].

To date, data on the impact of HZ and PHN on German patients’ QoL are scarce and limited to retrospective studies [7–9]. A recently published economic model evaluating the cost-effectiveness of a HZ vaccine in Germany mentioned the absence of utility data considering the impact on health related QoL caused by HZ and PHN specific for Germany as a limitation of their study [10]. Instead, they used data derived from a Canadian study which may not represent the real impact of HZ and PHN on QoL in Germany [11]. To remedy this and to provide relevant information for public healthcare decisions related to HZ prevention we undertook a prospective medium-term study of Germans aged 50 years or older consulting primary care physicians and diagnosed with acute HZ. The epidemiological outcomes, healthcare resource utilization and costs related to HZ are reported elsewhere for this study [12]. In this manuscript we focus on the impact of herpes zoster and postherpetic neuralgia on the quality of life of Germans aged 50 or above.

## Methods

### Study design

This prospective observational study was carried out in primary care practices in three regions of Germany (Fulda, Leverkusen, Marl). Primary care physicians (GPs, ophthalmologists and dermatologists) were invited to participate in a study over the period November 2010 to December 2014.

Patients meeting certain eligibility criteria were invited to participate in a cohort study aiming to estimate the proportion of HZ patients developing PHN and the economic burden and impact on health related QoL of both HZ and PHN. Patients were eligible if they were at least 50 years old at the time of first outpatient consultation presentation with HZ diagnosis, if they were deemed capable of completing the self-administered questionnaires used for measuring the impact and if they gave their written informed consent to participate.

The patient was presented with a booklet and asked to complete the QoL and pain assessment questionnaires during the initial consultation, with the physician providing assistance if required. The booklet contained further sets of questionnaires, which the patient was asked to complete and return to the physician or a central study site at the end of the follow-up period. Questionnaires were to be completed 15, 30, 60 and 90 days after rash onset.

Patients assessed as having PHN three months after rash onset (day 90) were asked to complete and return additional questionnaires at 120, 150 and 180 days after rash onset. Patients with persisting PHN at six months were asked to continue in the follow-up and complete and return additional questionnaires 210, 240 and 270 days (nine months) after rash onset. Otherwise, the follow-up of patients was not continued.

### Pain assessment questionnaire

HZ-related pain was assessed by item 3 of the ZBPI [13], asking the patient to indicate the ‘worst’ pain experienced over the previous 24 h on an 11-point Likert scale. The ZBPI scale was subdivided into four categories of pain: no pain (0); mild: (1–2); moderate (3–6); severe (7–10).

In addition to this measure of the ‘sensory’ dimension of pain, the ZBPI also contains items related to the ‘reactive’ dimension of pain intended to assess the impact of pain on the patient’s ADL. These items aim to assess the interference of pain on general activity, mood, walking ability, work, relations with others, sleep and enjoyment of life. Each of these seven items are assessed on an 11-point Likert scale with 0 = ‘does not interfere’ and 10 = ‘interferes completely’. The assessments of each item of function and activity were summarized and presented as a single ADL score by taking the mean of the seven items. If a response was missing for one item, the mean of the remaining six was used. If more than one item had no response, the summary score was coded as missing.

### QoL assessment

QoL was assessed by the standardized, generic EuroQoL five-dimension scale (EQ-5D) questionnaire [14, 15], which is designed for self-completion by the respondents and widely used across many disease areas to assess health states or outcomes of interventions. It consists of five items addressing mobility, self-care, usual activity, pain/discomfort and anxiety/depression and for each item there are three response categories: no problem, some problems and extreme problems. On the basis of the responses to these items, a summary weighted health utility index may be calculated using published preference weights derived from a sample of the general population (in this case German time-trade-off weights were used [15]). With these weights, the ‘worst health’ state imaginable has a utility index of - 0.205 whereas 1.000 represents ‘perfect health’. The EQ-5D also includes a visual analog scale (VAS) ranging from 0 to 100 on which the patients are asked to mark their global health state with 0 corresponding to ‘worst’ and 100 to ‘best imaginable health’.

### Case definitions

A case of HZ was defined as new unilateral pain (including allodynia and pruritus) accompanied by a unilateral rash and no alternative diagnosis. Clinically relevant PHN was defined as the presence of moderate or severe ‘worst’ pain (rated ≥ 3 on the ZBPI scale) persisting or appearing 90 days after onset of the HZ rash. The duration of PHN was defined as the time between HZ day 0 and the day of the first ZBPI assessment after day 90 in which the worst pain score was < 3. For PHN patients with no assessment in which the ZBPI worst pain score was < 3, the duration of PHN was censored at the day of their last assessment.

### Statistical analysis

Descriptive statistics are presented for demographic characteristics, and for the ZBPI pain severity scores. In addition, a repeated measures ANOVA model including terms for gender, age group and age group by time (window) interaction was fitted. The least squares means (LSmeans) estimates for time by age group was obtained from the ANOVA model. The PROC MIXED procedure in SAS was used to carry out the ANOVA, with all terms fitted as fixed effects [16–19]. An unstructured variance-covariance structure was assumed. This model was used to estimate the mean values of the ZBPI ADL scores and the EQ-5D scores in both HZ and PHN patients. An estimate statement within the PROC MIXED procedure in SAS was used to estimate the EQ-5D utility score in all HZ patients during the first 30 days and in all PHN patients for the period between the day 90 and day 120 assessments.

This procedure was used (as opposed to just taking the means of the observed data) in recognition that patients stop completing the questionnaires after a period of time, generally due to resolution of pain but possibly also for other reasons. An analysis of drop-outs in clinical trials of a candidate zoster vaccine (data not published) indicated that dropout was probably missing at random (as opposed to missing *completely* at random), i.e., drop-out was random conditional on the previous observed data [16–19]. This means that if the previous pain score was low or zero, the probability of drop-out was high. Therefore, the mean values based on observed data would potentially be biased towards higher (pain) or lower (QoL) values whereas the estimated LSmeans should be less biased. This analysis assumed normally distributed data although it is known that EQ-5D data are skewed. But, the mixed model is robust when distributions are not overtly skewed [20]. It must be noted, however, that LSmeans were not estimated after day 180 because the number of patients with persistent PHN was too small to use the model; after this time point simple observed means were reported.

In a post-hoc analysis not planned in the study protocol, the patients developing PHN were separated from the group of all the HZ patients and mean ZBPI and EQ-5D scores over the first three months were estimated for the group of HZ patients not developing PHN and the PHN patients separately.

The median duration of PHN and the proportion of patients with persisting PHN at nine months were estimated by the Kaplan-Meier product-limit method [21].

### Exploratory analyses

To describe the nature of the relationship between the dependent variable (HZ with and without PHN) and independent variables (risk or predictive factors), both univariate and multivariate logistic regression models were used in an exploratory analysis. In a previous epidemiological analysis of data from this study, the severity of acute HZ-related pain was found to be the only statistically significant predictive factor for PHN in a multivariate analysis including the following potential risk factors identified from previous published studies: age group; gender; pre-existing medical conditions (catch-all); diabetes; current immunosuppressive therapy; HZ severity at first visit, based on ZBPI ‘worst pain’ score [12]. Subsequently in this analysis, QoL variables were included in the model, i.e. the EQ-5D anxiety/depression scale, the EQ-5D VAS scale and the ZBPI ADL summary score, all recorded at the day 0 assessment. Using the backward elimination strategy (with *p*-value of 0.05 as the threshold to be kept in the model) and the method of maximum likelihood to estimate the parameters, the final regression equation was used to identify any statistically significant risk factors.

All analyses were carried out using SAS Version 9.2.

### Ethical requirements

The study was conducted in accordance with ethical principles having their origin in the Declaration of Helsinki, the principles of “good clinical practice” and all applicable regulatory requirements. The protocol was reviewed and approved by the ethics committees of the study areas (i.e. Landesärztekammer Hessen, Ethik-Kommission der Ärztekammer Nordrhein, and Ethik-Kommission der Medizinischen Fakultät der Universität Münster) and all enrolled patients gave written informed consent.

## Results

### Patient demographics and clinical characteristics

**Fig. 1:**
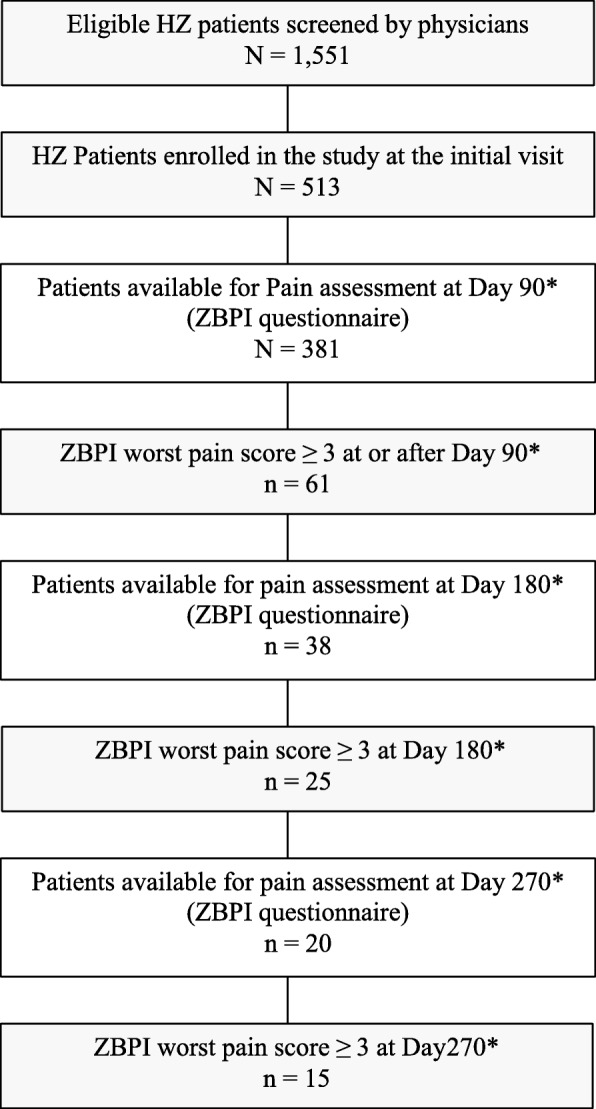
Flow chart**.** *after rash onset; HZ herpes zoster; ZBPI zoster brief pain inventory; N total number; n number

The demographics of the 513 HZ patients enrolled in the study are presented in Table 1. The mean and median ages were 67.8 and 69 years, respectively, and 63% were female. About 30% were still working, all of them in the age group younger than 65. Sixty-one patients experienced PHN, with the proportion increasing with rising age, from 10.6% in the group aged between 50 and 59 to 14.4% in those aged 80 years or older. 42 patients (8.2%) were reported by their study physician to have complications other than PHN. Eight patients (1.6%) had a cutaneous complication, 6 patients (1.2%) had an ocular complication, 9 (1.8%) had neurological complications (i.e. not including post-herpetic diagnosis) and 26 (5.1%) patients were reported as having other complications. The flow chart in Fig. 1 shows the course of the follow-up for the PHN patients, whereas for the remaining HZ patients follow-up was terminated at day 90.

**Table 1:** Patient demographics by age group of the 513 enrolled HZ patients

	50–59 y *N* = 142		60–64 y *N* = 59		65–69 y *N* = 67		70–79 y *N* = 182		≥ 80 y *N* = 63		Overall *N* = 513	
	n	%	n	%	n	%	n	%	n	%	n	%
Gender												
Male	48	33.8	25	42.4	23	34.3	62	34.1	32	50.8	190	37.0
Female	94	66.2	34	57.6	44	65.7	120	65.9	31	49.2	323	63.0
Employment												
(Self)-employed	115	81.0	29	49.2	–		–		–		144	28.1
Seeking work	6	4.2	–		–		–		–		6	1.2
Unemployed*	4	2.8	3	5.1	–		1	0.5	8		8	1.6
Retired	8	5.6	25	42.4	66	98.5	178	97.8	63	100	340	66.3
Missing	9	6.3	2	3.4	1	1.5	3	1.6	–		15	2.9
Age												
Mean	54.7		62.0		67.3		74.4		84.3		67.8	
SD	3.0		1.5		1.4		2.6		3.9		10.4	
Median	55		62		67		74		83		69	

*HZ* herpes zoster, *y* years old, *N* total number in each age group, n/%: number/percentage in each category, *SD* standard deviation, * unemployed, not seeking work

Approximately half of the patients (*N* = 252) reported having had symptoms before onset of the rash, mainly prodromal pain (75.8%, 191/252) and malaise (35.5%, 87/252). The mean delay between rash onset and the first consultation was 3.9 days (range 0–30). Of the 513 enrolled patients, only 381 returned evaluable ZBPI questionnaires on or after 90 days following rash onset and were thus evaluable for PHN assessment. The Kaplan-Meier estimate of the median duration of PHN was 276 days and 54.7% of the PHN patients were estimated as still suffering from PHN at day 270.

The rate of completion of evaluable questionnaires was high throughout the initial assessment period up to day 90, ranging from 86.7% at day 0 to 92.6% at day 30. The rate of completion for the day 90 (± 15 days) assessment was 88.5%. For the 61 PHN patients continuing in follow-up a drop in the completion rate was seen during the second three month period with 37 of the 61 subjects (60.7%) completing the assessment at day 180. Of the 25 PHN patients continuing in follow-up after day 180, 20/25 (80%) completed the assessment at day 270.

### All HZ patients

**Fig. 2:**
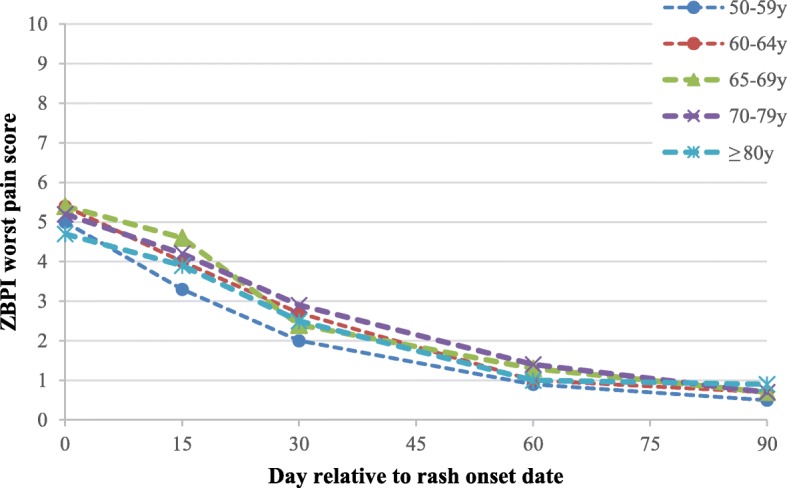
Mean ZBPI worst pain scores up to day 90 after HZ onset according to age group. ZBPI zoster brief pain inventory; HZ herpes zoster; y years old

The acute pain at day 0 was rated as moderate by the majority of the patients and the overall mean ZBPI ‘worst pain’ score was 5.1 (Fig. 2). After one month, the mean ZBPI ‘worst pain’ score had been reduced by half and at the end of the three months it was 0.7. The mean ZBPI ‘worst pain’ score was similar across the age groups at all time points.

**Fig. 3:**
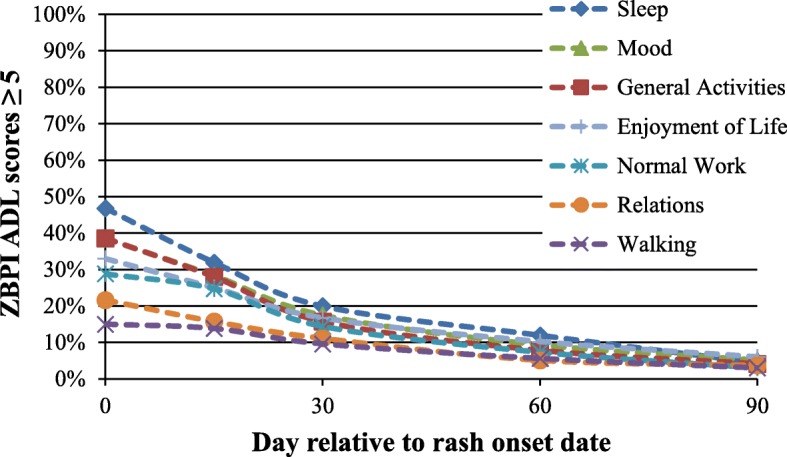
Percent of subjects with ZBPI ADL scores ≥ 5 after HZ onset by ADL individual items. ZBPI zoster brief pain inventory; HZ herpes zoster; ADL activities of daily living

Figure 3 presents the percent of ZBPI ADL individual item scores ≥ 5 over time. The item most affected was sleep with approximately 47% of subjects having a score ≥ 5 at Day 0, whereas walking ability was the least affected. The scores for each item diminished monotonically throughout the follow-up period (Fig. 3). Similarly, the mean ZBPI ADL scores as presented in Additional file 1: Figure S1, was highest at the Day 0 with an estimated LSmean (standard error [SE]) of 2.97 (0.12) and diminished monotonically over time to an LSmean of 0.66 (0.07) at day 90. This score did not show any great variation across the age groups at any time point (data not shown).

At day 0, the patients had an estimated LSmean (SE) EQ-5D utility index of 0.740 (0.012) with little variation across the age groups (Table 2). In each age group, monotonic increases of the mean score were observed over time but the absolute improvement over time was somewhat smaller in patients aged 80 years or older than in the younger age groups.

**Table 2:** Estimated LSMeans EQ-5D utility scores and EQ-5D VAS scores over 3 months by age group

Day	50–59 y *N* = 142		60–64 y *N* = 59		65–69 y *N* = 67		70–79 y *N* = 182		≥ 80 y *N* = 63		All *N* = 513	
	LSMean	SE	LSMean	SE	LSMean	SE	LSMean	SE	LSMean	SE	LSMean	SE
EQ-5D utility scores												
0	0.768	0.024	0.704	0.037	0.758	0.034	0.725	0.021	0.733	0.036	0.740	0.012
15	0.829	0.022	0.786	0.034	0.779	0.032	0.778	0.019	0.707	0.033	0.785	0.012
30	0.898	0.017	0.880	0.026	0.864	0.024	0.845	0.015	0.782	0.026	0.859	0.009
60	0.954	0.012	0.923	0.019	0.936	0.017	0.913	0.011	0.864	0.019	0.922	0.006
90	0.959	0.010	0.963	0.016	0.949	0.016	0.942	0.009	0.871	0.016	0.942	0.006
EQ-5D VAS scores												
0	61.0	2.1	58.7	3.1	59.3	2.9	61.0	1.8	58.3	3.1	60.2	1.1
15	70.7	1.9	65.7	3.0	64.9	2.7	62.8	1.7	59.7	2.8	65.2	1.0
30	76.5	2.0	73.2	3.0	72.5	2.9	69.0	1.7	69.3	3.0	72.1	1.0
60	85.8	1.8	80.7	2.8	84.1	2.6	80.1	1.6	77.0	2.7	81.9	0.9
90	88.9	1.7	85.5	2.7	87.2	2.5	86.1	1.5	81.2	2.6	86.4	0.9

*LS* least square, *HZ* herpes zoster, *EQ-5D* EuroQoL five-dimension scale, *VAS* visual analog scale, *N* total number in each age group, *SE* standard error, *y* years old

The LSmean EQ-5D VAS score was 60.2 at Day 0 and almost the same in each age group (Table 2). Over time the mean score increased monotonically in all age groups. At day 90, the lowest mean score and the smallest absolute improvement from day 0 was observed for those aged 80 years or older.

### PHN patients

At day 90, the PHN patients had a mean ZBPI ‘worst pain’ score of 4.4 which diminished monotonically until day 210, where it increased to 3.9 and then decreased again. This pattern is explained by the patients going off study in the course of the second three-month period until day 180, with the patients remaining in the study having a higher level of pain (data not shown in table). A similar pattern was observed for the ZBPI ADL score with an estimated LSmean of 2.90 (0.25) at day 90 and a mean of 2.43 at day 270. Neither the ZBPI ‘worst pain’ score nor the ZBPI ADL score showed any systematic relation with age.

### Predicting PHN

The results of the univariate analysis are presented in Additional file 1: Table S1. The only variables found to be significant on a univariate level were a history of diabetes mellitus (*p* = 0.0064), rash location back/spine (*p* = 0.0128) and all of the health related quality of life variables (see Additional file 1 for more details). We used an epidemiological model to estimate predictors of PHN in a multivariate analysis as the basis of our analysis of baseline QoL variables as predictors of PHN [12]. In the epidemiological model the only statistically significant variables were two separate variables for the severity of acute pain at the first visit, moderate and severe pain, respectively, with no/mild pain as the reference category. Including the QoL variables found to be significant in the univariate analyses, the EQ-5D anxiety/depression score and the EQ-5D VAS score, in the model together with the acute pain categories led to a final model with estimated statistically significant odds ratios of 5.00 (95% CI: 1.14–21.86) for developing PHN for patients with moderate pain versus those with no pain, 10.00 (95% CI: 2.32–43.14) for patients with severe pain versus those with no pain and 1.83 (95% CI: 1.04–3.24) for patients with a poor anxiety/depression score versus those with a score considered good.

51 patients (9.9%) reported having current emotional problems, i.e. stress or depression, as part of their pre-existing medical history. Of these 5 (9.8%) developed PHN (*p* = 0.3487). Out of 504 subjects with an EQ-5D assessment at Visit 1, 174 reported having moderate/extreme anxiety and depression. Of those 32 (18.4%) developed PHN (*p* = 0.0015).

### Post-hoc analyses

Given that the singularly most important predictors of developing PHN was the severity of pain at day 0, it was of interest to assess separately the ZBPI ‘worst pain’ scores and the EQ-5D utility scores for HZ patients who did develop PHN (“PHN patients”) and for HZ patients who did not develop PHN (“HZ patients”). At the day 0 assessment, the mean ZBPI ‘worst pain’ scores were 4.9 for HZ patients and 6.7 for PHN patients, diminishing monotonically over time in both groups (Table 3). For HZ patients, the mean ZBPI ‘worst pain’ score was negligible at day 90 whereas it remained high for PHN patients, 4.4.

**Table 3:** ZBPI worst pain scores and EQ-5D scores over the first 3 months for HZ patients according to their developing PHN or not

Day	HZ only (no PHN)			PHN		
	N	Mean	SD	N	Mean	SD
ZBPI ‘worst pain’ score						
0	394	4.9	2.8	50	6.7	2.2
15	396	3.6	2.7	51	6.5	2.0
30	417	2.1	2.6	56	5.7	2.4
60	410	0.7	1.7	54	4.9	2.1
90	393	0.1	0.8	58	4.4	1.5
EQ-5D score						
0	388	0.760	0.251	49	0.614	0.328
15	398	0.811	0.231	53	0.608	0.293
30	414	0.892	0.153	55	0.654	0.273
60	404	0.941	0.122	56	0.782	0.196
90	398	0.960	0.109	57	0.825	0.136

Note: The means presented in the table are the basic means of the observed data (not corrected by the least square model)

*N* number of evaluable questionnaires, *EQ-5D* EuroQoL five-dimension scale, *ZBPI* zoster brief pain inventory, *HZ* herpes zoster, *PHN* postherpetic neuralgia, *SD* standard deviation

For both groups, the EQ-5D utility score increased monotonically over the three-month period and by almost the same absolute amount but the PHN patients had clearly lower utility scores throughout (Table 3). The relation between acute pain and the utility score at day 0 is demonstrated in Table 4 for the ZBPI ‘worst pain’ and the ZBPI ‘average pain’ scores. Patients with severe ‘average pain’ had a mean utility score of 0.379 in contrast to 0.946 for those with no pain. Compared to patients whose PHN resolved during the second quarter after rash onset, patients with persistent PHN at or after day 180 had more severe ZBPI scores or lower utility scores already at day 0 (Additional file 1: Table S2).

**Table 4:** EQ-5D utility scores at Day 0 by ZBPI ‘worst pain’ and ‘average pain’ categories at Day 0

Pain type	Parameter	Pain category					Total
		No	Mild	Moderate	Severe	Missing	
Worst	N	38	87	207	102	3	437
	Mean	0.951	0.876	0.773	0.490	0.887	0.744
	SD	0.076	0.143	0.216	0.296	0.00	0.264
Average	N	42	140	217	34	4	437
	Mean	0.946	0.871	0.680	0.379	0.709	0.744
	SD	0.084	0.120	0.269	0.289	0.356	0.264

*N* number of evaluable questionnaires, *EQ-5D* EuroQoL five-dimension scale, *ZBPI* zoster brief pain inventory, *SD* standard deviation

## Discussion

This is the first prospective study of the impact of HZ and PHN on the QoL of primary care patients in Germany aged 50 or older. For the majority of the HZ patients, the period with pain and a considerable negative impact on QoL and interference with ADL was limited to a period of about one month after rash onset. The Kaplan-Meier estimate indicated that for about 50% of all HZ patients who developed PHN, the pain remained moderate or severe even nine months after rash onset. Neither the pain experienced nor the QoL scores showed any association with increasing age of the PHN patients.

Previous studies estimating utilities of HZ patients by means of the EQ-5D instrument have reported values ranging from 0.59 to 0.67 [11, 22–25], somewhat lower than the mean value of 0.740 at day 0 that we observed. Estimates of the utility score in PHN patients using the same instrument ranged from 0.59 to 0.67 [11, 26–28], substantially lower than the mean value of 0.825 at Day 90 estimated here.

If it is tentatively assumed that the mean EQ-5D utility score and VAS score estimated at day 90 for HZ patients who did not develop PHN corresponds to the normal value for people aged 50 or older (as there was not much age-related variation), an estimate of the impact of HZ and PHN may be obtained. With a normal utility score of 0.960, HZ had an immediate impact, i.e., a utility loss of 0.200 for those who did not develop PHN and of 0.346 for those who did. Relating the impact to the severity of acute pain, the estimated reduction of utility was 0.470 for patients with severe ‘worst pain’ and 0.581 for those with severe ‘average pain’. One estimate of the minimal important difference using the EQ-5D instrument is 0.074 [29], so the impact of HZ must be considered as substantial on average, even for those experiencing the least reduction of their QoL. These values are in line with other studies [24, 30–33].

Mean utility values were highly correlated with the mean pain scores over time, as has also been reported in other studies [11, 28]. This was seen both for PHN patients and for HZ patients who did not develop PHN. For the PHN patients, a clear reduction of pain and improvement of QoL was seen over the first 90 days but for patients with persistent PHN after six months the pain score reached a plateau about four months after rash onset with very small and gradual reductions thereafter and stable QoL values.

One study examining the general health states of people aged 75 or older in six European countries by means of the EQ-5D VAS instrument found a mean value for Germans of 60.6 (and a range across the countries from 60.2 to 72.0) [34]. This is intriguing given that in our study even patients with persistent PHN (those remaining in the group returning evaluable questionnaires after day 180) had mean VAS scores at day 90 equivalent to this ‘normal’ value. This finding underlines a need for further studies to establish well documented reference values for ‘normal, healthy’ individuals using the EQ-5D VAS scale.

The logistic regression indicated that the risk of developing PHN was higher in patients with more severe acute pain. Anxiety and depression were also determined to be risk factors for PHN. Previous studies have highlighted this association [35] and it has also been reported that individuals with intense HZ pain are at greater risk of anxiety and depression [36, 37]. As such it is difficult to know for certain the causal relationship, i.e., are subjects with chronic depression at a higher risk of PHN or does acute severe pain lead to more depression leading to an observed association between depression and pain? Results from this study would suggest that pre-existing, current stress or depression, reported during the medical examination, did not lead to an elevated increase in the risk of PHN, whereas results from the EQ-5D questionnaire suggested that anxiety and depression as measured at the initial visit post HZ onset was predictive of developing PHN. Dworkin et al. [35] reported that disease conviction, pain intensity, and state anxiety each made a unique contribution to discriminating subjects who did and did not develop PHN.

Previous studies [11, 38] have proposed a cut-off score of five or higher on the ZBPI ADL scores to distinguish between patients who experienced substantial interference and those experiencing little or no interference. In our study at day 0, 47% reported a score ≥ 5 for interference with sleep and about one third reported values ≥ 5 for general activity, mood and enjoyment of life. Drolet et al. noted that clinicians should be aware that patients who continue to experience herpetic pain are likely to encounter interference with ADL, anxiety or depression, and insomnia and will need careful attention and management of these conditions [11].

An estimate of the quality-adjusted life years (QALYs) gained by preventing one case of HZ in people aged 50 years or older in this study is approximately 0.03 to 0.04 QALY, depending on assumptions used. This is in the same range as reported by Kawai et al. [39], who estimated that based on the utility values reported in Ultsch et al. [7] the QALY loss due to HZ (including PHN) in Germany was approximately 0.03, 0.04 and 0.05 for 50–59, 60–74 and ≥ 75 year olds, respectively. This is equivalent to losing between 11 and 18 full days of perfect health for each HZ patient. On a population level, approximately 10,000 to 15,000 QALYs are lost annually due to HZ in individuals aged 50 years and older in the German population. HZ vaccination is the only known health care intervention that can prevent HZ and, consequently, PHN.

The enrolled patients were recruited among patients aged 50 years or older consulting primary care physicians for acute HZ. Out of the 1,551 patients screened (and included in the incidence estimation), only one third were enrolled in the study and one of the limitations of our study is that the patients who declined to enroll were not queried about their reasons for declining. The gender distribution of the enrolled patients was similar to that of all the screened patients but the age distribution was somewhat different as the enrolled patients tended to be younger than all the screened, with a higher proportion in the age group 50–59 years and a lower proportion of patients aged ≥ 80 years. And, according to the physicians’ assessment of the patients’ severity of pain at day 0, higher proportions of the enrolled patients had moderate or severe pain. The different age distribution of the enrolled compared to all the screened patients may have biased the proportion of PHN cases downward. In addition, as this study was carried out in a primary care physician setting the proportion of severe cases may be underestimated, as patients might have presented directly at the hospital instead of an ambulatory healthcare setting. This is supported by the proportion of HZ patients hospitalized which was 1.4% in our study compared with 3.2% in the study by Ultsch et al. [10]. Hence, the results may not be generalizable to the entire German population of HZ patients aged 50 years or older.

Another study limitation (and customary in all similar studies) is that the patients generally do not complete the questionnaires at the time of rash onset. The mean delay from rash onset to initial consultation was 3.7 days (with a range from 0 to 31 days) and this may lead to an underestimation of the overall pain and loss of QoL experienced during an episode of HZ as the impact seems worst at onset and diminishes gradually thereafter. As the delay between rash onset and the initial visit is greater than 72 h in most cases, the value of prescribing antiviral therapy becomes questionable [40]. It is also likely that the data present some information bias such as learning effects due to repeated testing over time with the same instruments. However, that potential bias must be balanced against the advantage of using the same instrument for easy assessment of the development over time. Furthermore, part of the change over time may rather reflect response shift (e.g., [41]), which is a potential attribution factor in any prospective study of the impact of disease on health related QoL.

One of the strengths of the study was that the patients were recruited at their first primary care consultation for acute HZ albeit with varying and probably unavoidable delays between rash onset and the visit. The patients were also followed over a relatively long time period so that all cases of HZ and most of the PHNs had resolved at the end of follow-up. Furthermore, we used well-validated measuring instruments, a clear and widely accepted definition of PHN and mixed modeling to diminish the potential bias due to questionnaire data missing not completely at random.

**Fig. 4:**
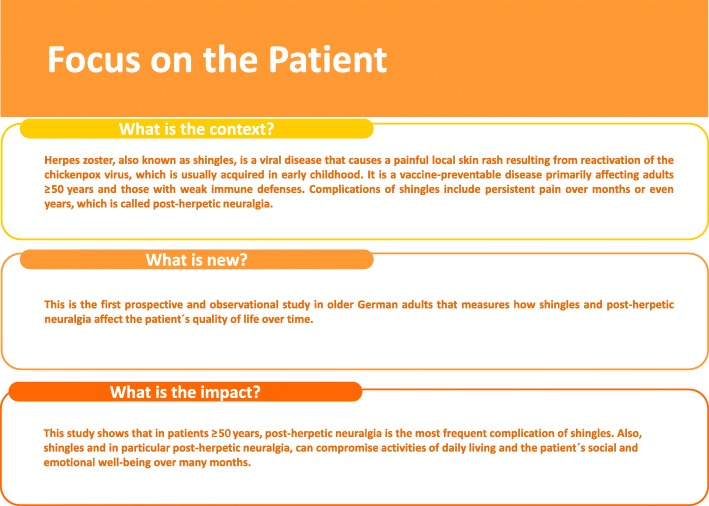
Lay language summary

A lay language summary contextualizing the results and potential clinical research relevance and impact is displayed in Fig. 4.

## Conclusion

Our study showed that HZ and PHN had a substantial impact on the patients’ QoL and ability to function in their normal activities and relations. For both groups, there was a close association in time between the development of pain and estimated QoL. The proportion of HZ patients developing PHN increased with rising age but the impact of PHN on ADL and patient QoL did not vary with age. For HZ patients, the absolute improvements in QoL over the three months following rash onset were lower for patients aged 80 years or older than for the other age groups.

## Additional file

Additional file 1: Discloses the mean zoster brief pain inventory activities of daily living interference score after herpes zoster onset by activities of daily living individual items and overall scores; the univariate predictive factor analysis and the risk factors for developing postherpetic neuralgia; and pain and EuroQoL five-dimension scale utility scores over time for patients with postherpetic neuralgia continuing at or after Day 180. (DOCX 42 kb)

## Abbreviations

ADL: Activities of daily living
EQ-5D: EuroQoL five-dimension scale
HZ: Herpes zoster
LSmeans: Least squares means
PHN: Postherpetic neuralgia
QALY: Quality adjusted life years
QoL: Quality of life
SE: Standard error
VAS: Visual analog scale
VZV: Varicella-zoster virus
ZBPI: Zoster brief pain inventory
