# Supplementary material for: Impact of herpes zoster and postherpetic neuralgia on the quality of life of Germans aged 50 or above
Source: BMC Infect Dis. 2018 Oct 3;18:496. doi: 10.1186/s12879-018-3395-z (PMC6169019; doi:10.1186/s12879-018-3395-z)
Supplement: Supplementary file 1 — Discloses the mean zoster brief pain inventory activities of daily living interference score after herpes zoster onset by activities of daily living individual items and overall scores; the univariate predictive factor analysis and the risk factors for developing postherpetic neuralgia; and pain and EuroQoL five-dimension scale utility scores over time for patients with postherpetic neuralgia continuing at or after Day 180. (DOCX 42 kb) [file 12879_2018_3395_MOESM1_ESM.docx]

Additional file 1

Impact of herpes zoster and postherpetic neuralgia on the quality of life of Germans aged 50 or above

Desmond Curran, Ruprecht Schmidt-Ott, Ulf Schutter, Jörg Simon, Anastassia Anastassopoulou, Sean Matthews

BMC Infectious Diseases

**Figure S1. Mean ZBPI ADL interference score after HZ onset by ADL individual items and overall scores**

ZBPI: zoster brief pain inventory; HZ: herpes zoster; ADL: activities of daily living

**Univariate predictive factor analysis of postherpetic neuralgia (PHN)**

A logistic regression model was used to investigate risk factors for PHN. Risk factors included were age, gender, herpes zoster (HZ) related complications, symptoms before the rash (i.e. prodromal pain), timing of treatment with antivirals, current immunosuppressive therapies (i.e. oral or parenteral corticosteroids, cytostatics/chemotherapy treatment, monoclonal and polyclonal antibodies), pre-existing medical histories (i.e. diabetes mellitus, current emotional problems (stress or depression), HZ location (i.e. head and neck; back and spine; chest and abdomen; upper limbs; lower limbs) and health related quality of life variables (i.e. Zoster Brief Pain Inventory (ZBPI) pain severity, ZBPI activities of daily living, EQ-5D anxiety and depression score and the EuroQoL five-dimension scale (EQ-5D) visual analog scale score).

The only variables found to be significant on a univariate level were a history of diabetes mellitus (p=0.0064), rash location back/spine (p=0.0128) and all of the health related quality of life variables.

These results need to be interpreted with caution as (1) there are small numbers of subjects in various categories in association with a relatively small number of PHN cases overall and (2) there is no adjustment for multiple comparisons.

**Table S1. Risk factors for developing PHN**

|  | | | | | **95% CI of odds ratio** | |  |
| --- | --- | --- | --- | --- | --- | --- | --- |
| **Category** | **Component** |  | **N1/N2 (%)** | **Odds ratio** | **LL** | **UL** | **p-value** |
| **Epidemiology variables** | Age group | 50-59** | 15/142 (10.6) | . | . | . | . |
|  |  | 60-69 | 17/126 (13.5) | 1.320 | 0.630 | 2.768 | 0.4615 |
|  |  | 70-79 | 20/182 (11.0) | 1.045 | 0.515 | 2.123 | 0.9026 |
|  |  | >80 | 9/63 (14.3) | 1.411 | 0.582 | 3.421 | 0.4458 |
|  | Gender | Male** | 18/190 (9.5) | 1.467 | 0.820 | 2.626 | 0.1968 |
|  |  | Female | 43/323 (13.3) | . | . | . | . |
|  | HZ related complications at initial visit | No** | 52/468 (11.1) | 2.207 | 0.958 | 5.082 | 0.0629 |
|  |  | Yes | 8/37 (21.6) | . | . | . | . |
|  | Patient experiences any symptoms before the rash | No** | 26/251 (10.4) | 1.350 | 0.784 | 2.324 | 0.2796 |
|  |  | Yes | 34/252 (13.5) | . | . | . | . |
|  | Prodromal pain | No** | 32/312 (10.3) | 1.503 | 0.874 | 2.587 | 0.1410 |
|  |  | Yes | 28/191 (14.7) | . | . | . | . |
|  | Timing of treatment with antivirals | <3** | 8/102 (7.8) | 1.448 | 0.605 | 3.465 | 0.4052 |
|  |  | ≥3 | 18/164 (11.0) | . | . | . | . |
|  | Current immunosuppressive medications | No | 55/450 (12.2) | 1.079 | 0.367 | 3.174 | 0.8900 |
|  |  | Yes** | 4/35 (11.4) | . | . | . | . |
|  | HZ related complications at initial visit | No** | 52/468 (11.1) | 2.207 | 0.958 | 5.082 | 0.0629 |
|  |  | Yes | 8/37 (21.6) | . | . | . | . |
|  | Pre-existing medical condition | Yes | 15/187 (8.0) | 1.862 | 1.005 | 3.448 | 0.0481 |
|  |  | No** | 44/315 (14.0) | . | . | . | . |
|  | Diabetes | No** | 43/427 (10.1) | 2.422 | 1.282 | 4.575 | 0.0064 |
|  |  | Yes | 16/75 (21.3) | . | . | . | . |
|  | Current emotional problems, stress | No** | 54/451 (12.0) | 0.799 | 0.304 | 2.099 | 0.6491 |
|  |  | Yes | 5/51 (9.8) | . | . | . | . |
| **Rash Location** | Head and neck | No Rash** | 48/396 (12.1) | 0.906 | 0.473 | 1.737 | 0.7669 |
|  |  | Rash | 13/117 (11.1) | . | . | . | . |
|  | Upper limbs | No Rash** | 55/475 (11.6) | 1.432 | 0.573 | 3.579 | 0.4425 |
|  |  | Rash | 6/38 (15.8) | . | . | . | . |
|  | Back and spine | No Rash** | 20/246 (8.1) | 2.050 | 1.165 | 3.609 | 0.0128 |
|  |  | Rash | 41/267 (15.4) | . | . | . | . |
|  | Chest and abdomen | No Rash** | 42/322 (13.0) | 0.736 | 0.415 | 1.308 | 0.2965 |
|  |  | Rash | 19/191 (9.9) | . | . | . | . |
|  | Lower limbs | No Rash** | 50/456 (11.0) | 1.942 | 0.945 | 3.991 | 0.0710 |
|  |  | Rash | 11/57 (19.3) | . | . | . | . |
| **Health**  **Related**  **Quality of Life**  **Variables** | ZBPI  pain score | Mild** | 2/103 (1.9) | . | . | . | . |
|  |  | Moderate | 23/215 (10.7) | 6.049 | 1.398 | 26.175 | 0.0160 |
|  |  | Severe | 35/184 (19.0) | 11.862 | 2.790 | 50.428 | 0.0008 |
|  | ZBPI  activities of daily living score | Good** | 18/258 (7.0) | 2.704 | 1.514 | 4.832 | 0.0008 |
|  |  | Poor | 43/255 (16.9) | . | . | . | . |
|  | EQ-5D anxiety/depression score | Good** | 28/328 (8.5) | 2.414 | 1.400 | 4.164 | 0.0015 |
|  |  | Poor | 32/174 (18.4) | . | . | . | . |
|  | EQ-5D  VAS Score | Good** | 18/274 (6.6) | 3.120 | 1.746 | 5.577 | 0.0001 |
|  |  | Poor | 43/239 (18.0) | . | . | . | . |

N1: number of subjects with PHN; N2: total number of subjects in category; LL: lower limit; UL: upper limit; CI: confidence interval; ZBPI: zoster brief pain inventory ; HZ: herpes zoster; PHN: ; 95% CI of odds ratio: wald 95% confidence interval of odds ratio; EQ-5D: EuroQoL five-dimension scale ; VAS: visual analog scale; **reference category

**Table S2. Pain and EQ-5D utility scores over time for patients with PHN continuing at or after Day 180.**

| **Day** | **Worst pain score** | | |  | **EQ-5D utility score** | | |
| --- | --- | --- | --- | --- | --- | --- | --- |
|  | **N** | **Mean** | **SD** |  | **N** | **Mean** | **SD** |
| 0 | 20 | 6.9 | 2.2 |  | 21 | 0.594 | 0.336 |
| 15 | 22 | 6.5 | 2.0 |  | 21 | 0.586 | 0.286 |
| 30 | 23 | 5.7 | 2.3 |  | 23 | 0.650 | 0.275 |
| 60 | 23 | 4.9 | 1.7 |  | 24 | 0.787 | 0.189 |
| 90 | 23 | 4.6 | 1.3 |  | 23 | 0.850 | 0.068 |
| 120 | 24 | 4.2 | 1.6 |  | 23 | 0.805 | 0.170 |
| 150 | 23 | 4.0 | 1.8 |  | 23 | 0.854 | 0.145 |
| 180 | 22 | 3.8 | 1.3 |  | 22 | 0.866 | 0.060 |
| 210 | 21 | 3.9 | 1.7 |  | 21 | 0.866 | 0.060 |
| 240 | 18 | 3.8 | 1.7 |  | 19 | 0.862 | 0.053 |
| 270 | 22 | 3.2 | 2.3 |  | 21 | 0.835 | 0.162 |

PHN: postherpetic neuralgia ; N: number of evaluable questionnaires ; EQ-5D: EuroQoL five-dimension scale ; SD: standard deviation
